# Accelerated Metabolomic Aging and Its Association with Social Determinants of Health in Multiple Sclerosis

**DOI:** 10.1101/2025.01.29.25321260

**Published:** 2025-02-03

**Authors:** Fatemeh Siavoshi, Rezvan Noroozi, Gina Chang, Vinicius A. Schoeps, Matthew D. Smith, Farren B.S. Briggs, Jennifer S. Graves, Emmanuelle Waubant, Ellen M. Mowry, Peter A. Calabresi, Pavan Bhargava, Kathryn C Fitzgerald

**Affiliations:** 1 Department of Neurology, Johns Hopkins University School of Medicine, Baltimore, MD, USA; 2 Department of Neurology, Children's Hospital of Philadelphia, Philadelphia, PA, USA; 3 Department of Neurology, University of California, San Francisco, San Francisco, CA, USA; 4 Department of Epidemiology, Johns Hopkins University School of Public Health, Baltimore, MD, USA; 5 Department of Public Health Sciences, University of Miami Miller School of Medicine, Miami, FL, USA; 6 Department of Neurosciences, University of California, San Diego, San Diego, CA, USA.

## Abstract

Biological age (BA), shaped by genetics, lifestyle, and environmental exposures, reflects physiological changes and mediates the effects of social determinants of health (SDoH) in diseases other than multiple sclerosis (MS). We investigated BA acceleration through metabolic profiling in people with MS (PwMS) and its association with SDoH, measured by the area deprivation index. Accelerated BA was observed in PwMS compared to healthy controls in independent adult and pediatric-onset MS cohorts. Greater social deprivation correlated with greater BA acceleration in PwMS. These findings highlight BA as a potential mediator linking SDoH to MS outcomes and a target to reduce health disparities.

## Introduction

Chronological age (CA), determined by birth date is associated with multiple sclerosis (MS) progression and therapeutic response^1^. However, disease progression varies among people with MS (PwMS) of the same CA due to genetic and environmental interactions. Biological age (BA), a measure of physiological aging and cellular health, may provide a more accurate representation of these differences than CA, reflecting an individual's health status more effectively.

Social and environmental conditions play a crucial role in shaping biological patterns. Social determinants of health (SDoH)—non-medical factors like living conditions— influence physical and mental health outcomes, including those in PwMS^2,3^. In the general population, unfavorable SDoH are linked to earlier onset and greater severity of age-related diseases, suggesting they may accelerate biological aging^4,5^. This accelerated aging could also mediate the impact of SDoH on MS progression, highlighting the importance of exploring this relationship in MS.

This study compares BA, estimated through metabolomic profiles, in PwMS and healthy controls (HCs), and examines its association with SDoH in PwMS. While BA is commonly assessed using biomarkers such as telomere length, DNA methylation, or clinical laboratory values, metabolomics provides additional insight by reflecting the downstream products of biological processes influenced by the genetic and, notably, environmental exposures relevant to SDoH^6–9^.

## Methods

### Study participants

Data were pooled from IRB-approved research studies at two sites where blood samples were collected for metabolomic analyses: the Johns Hopkins MS Precision Medicine Center of Excellence (JHU) and the pediatric MS Center at the University of California, San Francisco (UCSF). The JHU cohort included PwMS or HCs, and the UCSF cohort included HCs and those with pediatric-onset MS (POMS) or clinically isolated syndrome (CIS) meeting the 2017 McDonald criteria, with onset before age 18 and within four years of symptom onset.

### Measures of social determinants of health

We used the area deprivation index (ADI) as the primary SDoH indicator at the census block group level at the national level. The ADI, a composite index linked to health outcomes in the general population and PwMS, incorporates 17 socioeconomic status (SES) measures (e.g., income disparity, vehicle access, occupational composition, single-parent households, and English proficiency)^2,10,11^. The national ADI ranges from 1 (least disadvantaged) to 100 (most disadvantaged). ADI analyses included only PwMS in the JHU cohort, as geocodes required to derive census block groups were available exclusively for these participants.

### Metabolomic measurements

All blood samples were processed within 3 hours and stored at −80°C following a standardized protocol. Metabolomic profiling for both cohorts was performed using an untargeted platform by Metabolon Inc. (Durham, NC) as detailed previously^12^. After thawing, samples were derivatized and analyzed using gas chromatography-mass spectrometry or liquid chromatography-tandem mass spectrometry. Mass spectra were compared to a reference library for metabolite identification, and relative abundances were calculated from the area under the curve. Profiling was conducted in five batches for JHU and two for UCSF.

### Statistical analysis

#### Preprocessing and quality control

We applied consistent quality control and harmonization across cohorts, excluding metabolites with >20% missing values per batch. Missing values in the remaining metabolites were imputed using k-nearest neighbors (k = 10) and log-transformed. Metabolomic profiling identified 332 and 646 metabolites across JHU and UCSF batches, respectively. Within-site batch effects were harmonized using ComBat, an empirical Bayes method previously applied to metabolomics by our team^14^. Finally, data were normalized via Z-scores.

#### Metabolomic age calculation

Metabolomic age (MA) was calculated using a previously published model developed based on healthy individuals aged 18–75 years from the INTERVAL study (n=11,977) in Cambridge, UK, with metabolomic profiling performed by Metabolon Inc. using methods similar to our cohorts^13^. The MA model included 826 metabolites (678 endogenous and 148 xenobiotics) and had two versions: Model A (826 metabolites) and Model B (678 metabolites). MA was calculated by applying the intercept, sex coefficient, and the sum of metabolites obtained by multiplying each metabolite's level by its corresponding coefficient for matching metabolites—332 in the JHU and 563 in the UCSF dataset—from Model A, as this model with both endogenous and xenobiotics, accounted for more environmental exposures.

#### Descriptive and analytical models

Descriptive analyses summarized categorical variables as frequencies and continuous variables as means or medians. We assessed MA in PwMS versus HCs using cross-sectional and longitudinal analyses. Age acceleration, defined as the difference between MA and CA, was the primary effect. A generalized linear model was used for between-group comparisons in the UCSF cohort, with one sample per individual. In the JHU cohort, with multiple samples for 195 individuals (50.13%), the linear mixed-effects (LME) model accounted for within-person correlation. For the subset of participants with multiple samples and follow-up exceeding one year, we fit an LME model assessing if PwMS exhibited a faster rate of biological aging compared to HCs, including group, time, and interaction term; the group-time interaction reflects a measure of whether biological aging was increased in PwMS relative to HC.

The association between age acceleration and ADI in PwMS was analyzed cross-sectionally using linear regression within the JHU cohort, with additional exploration of whether this relationship varied by race. Analyses were performed using R version 4.4.1, with a significance threshold of P < 0.05.

## Results

### Demographics characteristics

The JHU cohort included 389 participants contributing 683 samples: 66 HCs (142 samples, 20.79%) and 323 PwMS (541 samples, 79.21%). The median age of participants was 45.09 years (interquartile range [IQR]: 35.13–53.77 years), with 72.62 % female (n = 496 samples) and 81.99% identifying as White (n = 560 samples). The UCSF cohort comprised 131 participants: 67 HCs (51.15%) and 64 with POMS/CIS (48.85%). The median age was 14.7 (IQR=12.35–16.32) years, with 47.33% female (n=62). The detailed characteristics of the participants for the cohorts are shown in Table 1.

### Comparison of metabolomic age acceleration

**Figure 1.**
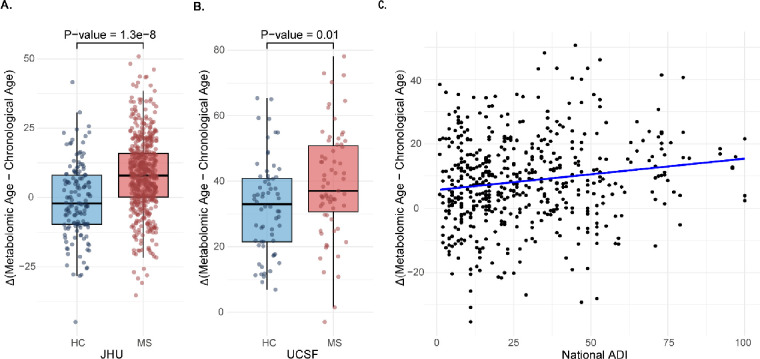
Metabolomic aging in multiple sclerosis and its relationship with social determinants of health. Comparison of metabolomic age acceleration (metabolomic age - chronological age) between individuals with Multiple Sclerosis (MS) and Healthy Controls (HC) across cohorts. **A.** JHU cohort **B.** UCSF cohort **C.** Association of social deprivation with metabolomic age acceleration in people with MS in the JHU cohort

Cross-sectionally, adult PwMS exhibited a pronounced greater age acceleration, with a mean difference of 9.48 years (95% CI: 6.29, 12.67) compared to HCs (p < 0.0001). Participants with POMS/CIS demonstrated a greater average age acceleration of 7.05 years (95% CI: 1.71, 12.40) compared to HCs (p = 0.01) (Figures 1A and 1B).

Longitudinal analysis in adult participants with follow-up data (n=173; average follow-up of 4.57 years) revealed a faster biological aging rate in PwMS than in HCs, with an average increase of 1.01 years of MA per chronological year (95% CI: 0.00, 2.00; p=0.04).

### Correlation of metabolomic age acceleration with ADI

ADI ranged from 1 to 100 with a mean (SD) of 27.1 (20.7). Increasing social deprivation was associated with greater age acceleration. In PwMS, a 10-percentile increase in ADI was associated with a 0.71-year (95% CI: 0.17, 1.25) increase in age acceleration (p=0.01) after accounting for CA, gender, race, MS subtype, and MS therapy (Figure 1C). Results were consistent in analyses stratified by race.

## Discussion

We demonstrated accelerated metabolomic aging, both cross-sectionally and longitudinally in PwMS compared to HCs, aligning with previous research using other BA markers^8,15^. Age acceleration was evident even in pediatric-onset MS, suggesting it is a core feature of MS, independent of disease duration or prolonged disease-modifying therapy exposure. We also demonstrated that greater social deprivation was associated with greater biological aging in PwMS, providing one candidate biological rationale linking socioeconomic factors with worse disease outcomes.

Chronic inflammation, immune dysregulation, and cellular stress in MS likely drive aging-related processes, including mitochondrial dysfunction and DNA damage, contributing to the higher age-related comorbidities, such as cardiovascular disease and cognitive decline, further exacerbated by social deprivation^1,16–18^. The ADI reflects aspects of deprivation that contribute to chronic stress, limited health resources, and disrupted dietary patterns, expediting biological aging. Lower SES is associated with worse MS outcomes, and our findings provide biological insight into this relationship^2,3^. This aligns with research in cardiovascular disease, where social factors influence outcomes via aging acceleration^5,19^. These observations underscore the importance of addressing social deprivation in MS and suggest that MA acceleration could be a measurable target for interventions to reduce health disparities, warranting future studies.

This study has certain limitations. Ideally, MA would be calculated using the full set of metabolites from the MA model, but the retrospective design limited the metabolites available, resulting in differences in the metabolites used for each cohort. This prevented direct comparison of MA values across cohorts with varying demographic and clinical characteristics. To maintain consistency, we used the same metabolomic clock for both cohorts, despite it being developed with adult data. Longitudinal and ADI data were unavailable for the UCSF cohort, limiting the generalizability of ADI-associated findings and preventing longitudinal analyses. While the ADI served as a proxy for SES, it is an ecologic variable at the neighborhood level and does not fully capture other SDoH, like social support, and lifestyle factors, which may influence biological aging.

## Conclusion

We demonstrated accelerated biological aging in PwMS and its association with social disadvantage, offering insights into how socioeconomic factors may influence MS severity and progression. Future studies with larger cohorts and broader SDoH assessments will help further validate these findings and unravel specific mediators of accelerated aging.

## Figures and Tables

**Table 1.** Characteristics of included study participants

Variable	JHU^1^		UCSF^2^	

	HC	MS	HC	MS

No. of samples	142	541	64	67

No. of participants	66	323	64	67

No. of samples per participant (mean)	2.15	1.67	1.00	1.00

Follow-up time, mean (SD)	2.38 (2.04)	4.76 (2.74)	-	-

Age, y, mean (SD)	42.54 (12.83)	45.35 (11.90)	14.30 (2.52)	14.20 (2.72)

Age range (min, max)	22, 84	18, 72	8, 18	7, 18

Female gender, n (%)	106 (74.65)	390 (72.09)	30 (46.87)	32 (47.76)

Race				
- Black or African American, n (%)	21 (14.79)	70 (12.94)	3 (4.69)	5 (7.46)
- White, n (%)	105 (73.94)	455 (84.10)	56 (87.50)	56 (83.58)
- Others, n (%)	16 (11.27)	16 (2.96)	5 (7.81)	6 (8.96)

Progressive MS, n (%)	-	152 (28.10)		

DMT Status^3^				
- No DMT, n (%)	-	141 (26.06)	-	-
- Mild efficacy, n (%)	-	178 (32.90)	-	-
- Moderate efficacy, n (%)	-	54 (9.98)	-	-
- High efficacy, n (%)	-	168 (31.05)	-	-

1. JHU: Johns Hopkins MS Precision Medicine Center of Excellence

2. UCSF: University of California, San Francisco

3. Disease-modifying therapy (DMT) status reflects the treatment participants were receiving at the time of the blood draw for the JHU cohort, classified as high-efficacy (natalizumab, ocrelizumab, rituximab, daclizumab), moderate-efficacy (fingolimod, dimethyl fumarate), mild-efficacy (glatiramer acetate, interferon beta, azathioprine, teriflunomide, mycophenolate mofetil), or not on DMT.

## Data Availability

The data that support the findings of this study are available from the corresponding author upon reasonable request.
